# Report of a Case of Carbuncle

**Published:** 1892-03

**Authors:** W. B. Cox

**Affiliations:** Landsford, S. C.


### REPORT OF A CASE OF CARBUNCLE.

BY W. B. COX, M. D., LANDSFORD, S. C.

Thomas R., aged about 40, rode to a town distant about 15 miles, indulged freely in whisky, returned and continued to indulge freely for about ten days, when he sent for me. I found him prostrated, with no appetite, slight elevation of temperature, suffering with insomnia and some gastric irritability. On the left buttock he had a circumscribed swelling about 4 1-2 inches in diameter, which he told me he had discovered shortly after his return from town, to which he had ridden in a wagon with a board thrown across the wagon body for a seat. The swelling was giving him considerable pain. He was still drinking freely.

I gave him a mercurial, to be followed in four or five hours with a saline of sulph. magnesia, injected sulph. morph., gr. 1-4, and sulph. atropia, gr. 1-150, in proximity to the swelling, smeared the swelling with belladonna ointment, over which I ordered that poultices of hot mush be kept continually applied, and gave him 15 grs. chloral, to be repeated every hour until sleep was produced. I did not withdraw the whisky altogether, but restricted him to 1 1-2 oz. every two hours. I allowed him a generous diet of sweet milk, poached eggs, gruels, etc., and ordered that he drink *ad libitum* chicken broth highly seasoned with pepper. I find that chicken broth—indeed, any other kind of broth—seasoned highly with pepper and taken as hot as can be borne, very agreeable to the stomach after a *debauch.*

The next day the mercurial and saline were repeated. On the third day I observed the cribiform openings so peculiar to carbuncles.

By this time his secretions were in a better condition, and I gave him the following:

℞ Mur. tinct. ferri, ℥ ss.

Sulph. quiniæ, gr. xlviii.

Liq. strychninæ, ʒ i.

Aquæ, q. s., ℥ iii. M.

Sig. Teaspoonful well diluted with water every six hours.

In my gynæcological bag I chanced to have a preparation containing equal parts of carbolic acid, tincture of iodine and glycerine. With absorbent cotton wrapped around the end of a straw I made an application of this preparation to the whole carbuncular mass, boring well into each one of the cribiform openings. I then made three or four successive applications of tincture of iodine, extending the applications some distance beyond the circumference of the swelling. As a dressing, I used iodoform gauze and absorbent cotton secured by a roller bandage drawn moderately tight.

These applications and dressings were renewed every third day. The patient improved rapidly and was dismissed on the fourteenth day from the time I first saw him.

If the carbuncular nature of the swelling had been recognized, and the carbolic acid and iodine treatment had been instituted at first, I believe my patient would have recovered sooner.

The treatment in this case is nothing new. My only reason for reporting it is that some one who has had no experience with it may be induced to try it. Some prefer using the carbolic acid hypodermically, others locally. I prefer the latter. The latter is certainly less painful, and pr. bably just as efficacious. But in the treatment of carbuncle, as well as in all other inflammatory conditions, we must not neglect constitutional treatment. Secretion and excretion must be kept as near as possible within physiological limits; that is, we must keep in mind the nutrition of the patient.

